# Pancreatic cancer complicated with a giant peritoneal loose body: case report and literature review

**DOI:** 10.3389/fonc.2026.1684923

**Published:** 2026-03-16

**Authors:** Runjie Hou, Mingyue Du, Jing Guo, Kaimeng Wang, Yuan Zhang, Yilong Wang, Pengcheng Liu, Fei Guo, Jijun Wang

**Affiliations:** 1 Department of Gastrointestinal Surgery, Baotou Central Hospital, Baotou, China; 2 Baotou Medical College, Baotou, China; 3 Department of General Surgery, Xiwu Banner People's Hospital, Xilinhot, China; 4 Department of Anesthesiology, Baotou Central Hospital, Baotou, China

**Keywords:** diagnostic and therapeutic strategy, differential diagnosis, giant peritoneal loose body, laparoscopic surgery, pelvic tumor

## Abstract

This paper reports a case of an 82-year-old male with a giant peritoneal loose body (GPLB) complicated by pancreatic head cancer. The patient was admitted for upper abdominal pain and jaundice. CT revealed a 5.5 × 5.6 cm oval low-density mass on the right side of the pelvis with a central high-density calcified focus, initially diagnosed as a gastrointestinal stromal tumor. During surgery, a yellow free mass measuring 6 × 6 × 5 cm was found in the pelvic cavity; pathology confirmed that it was a peritoneal loose body. Grossly, it was yellow and ovoid, with central calcification on the cut surface; microscopically, necrotic adipose tissue and hyalinized collagen fibers were visible. Meanwhile, we comprehensively reviewed and analyzed 32 previously reported cases of GPLB, summarizing the disease characteristics, clinical manifestations, formation mechanisms, diagnostic approaches, and treatment strategies. We propose a hypothesis-generating observation that abnormal liver and kidney function may accelerate the growth rate of giant peritoneal loose bodies. We also recommend that surgical removal be performed regardless of whether symptoms are present, with laparoscopy as the preferred procedure. This study aims to improve clinicians’ understanding of this disease and reduce misdiagnosis.

## Case report

**Figure 1:**
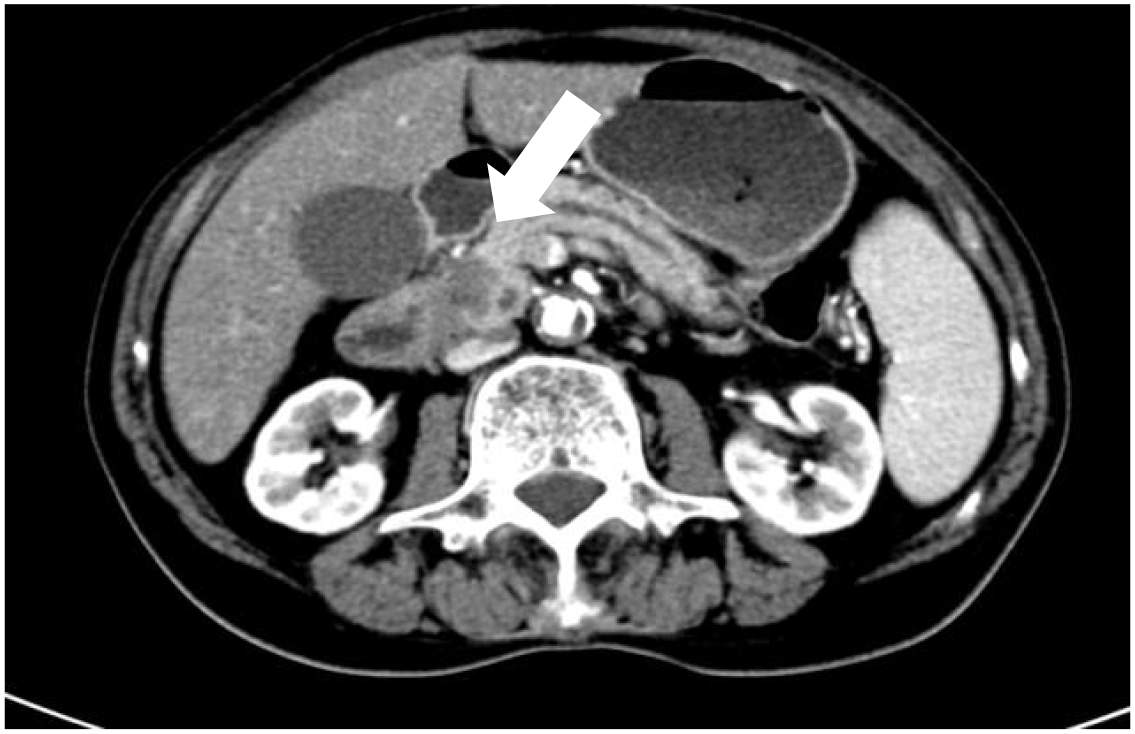
Contrast-enhanced CT shows an occupying lesion in the pancreatic head invading the duodenum.

**Figure 2:**
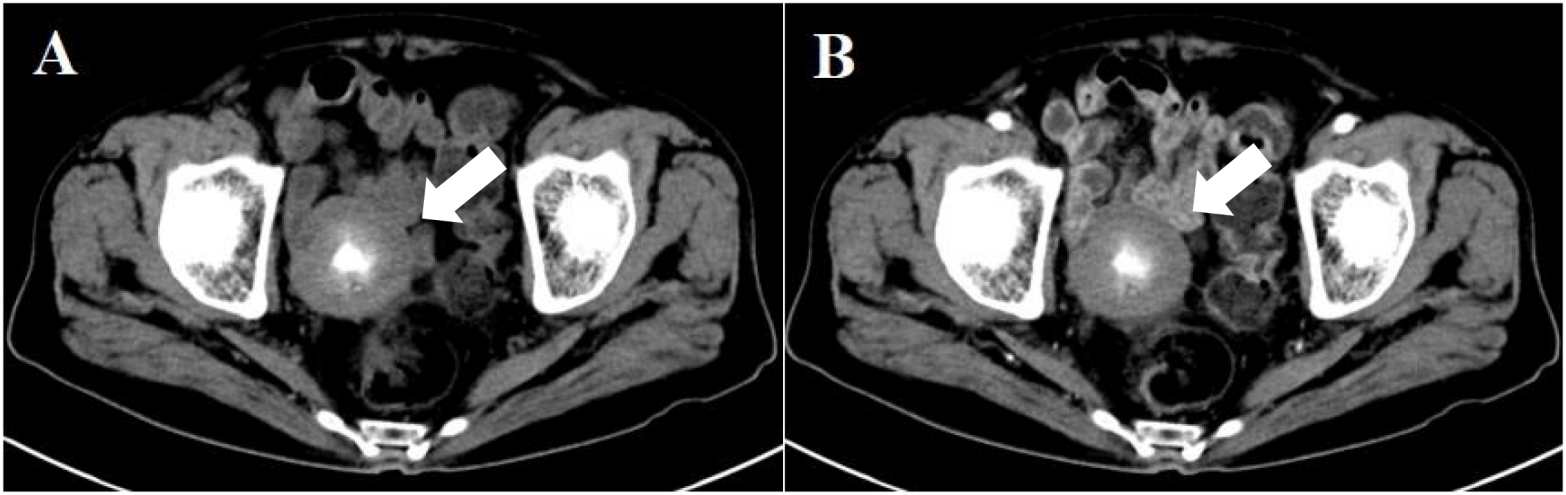
Computed tomography reveals a quasi-round mass (indicated by the white arrow), with soft-tissue density at the periphery and irregular central calcification **(A)**. Contrast-enhanced CT shows no enhancement of the quasi-round mass **(B)**.

**Figure 3:**
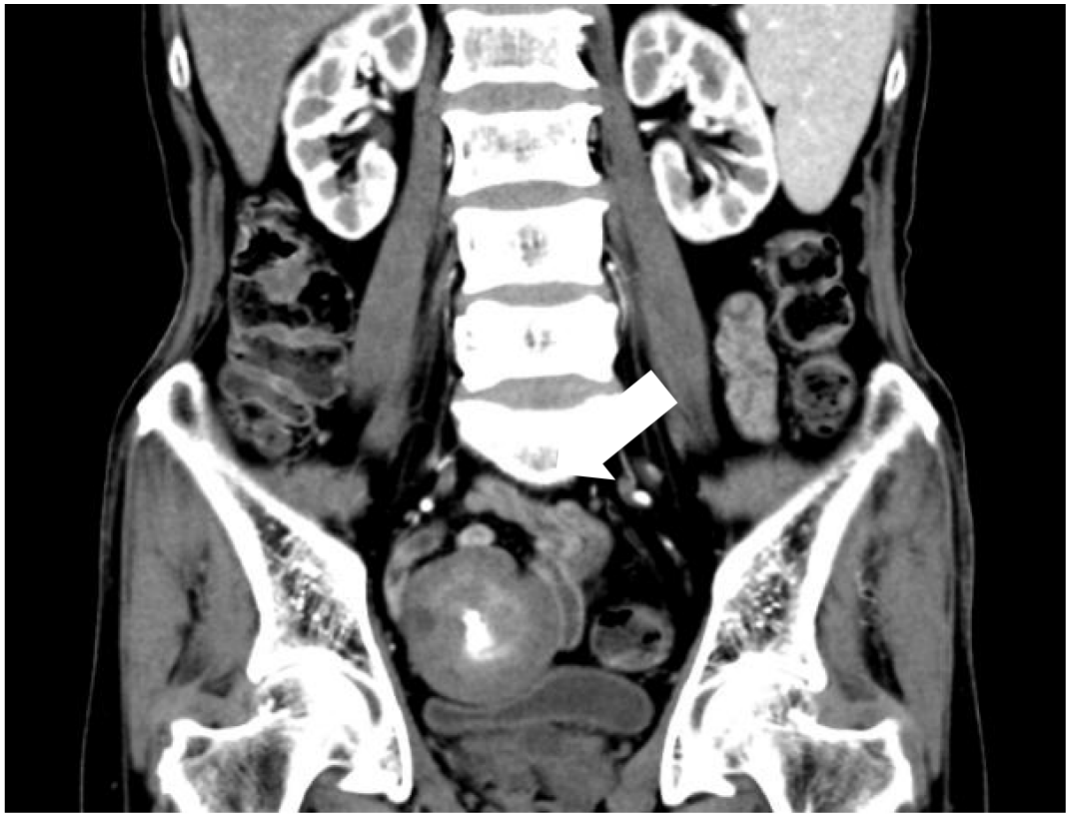
Computed tomography shows that the mass (indicated by the white arrow) demonstrates no enhancement and is located in the pelvis, compressing the bladder.

**Figure 4:**
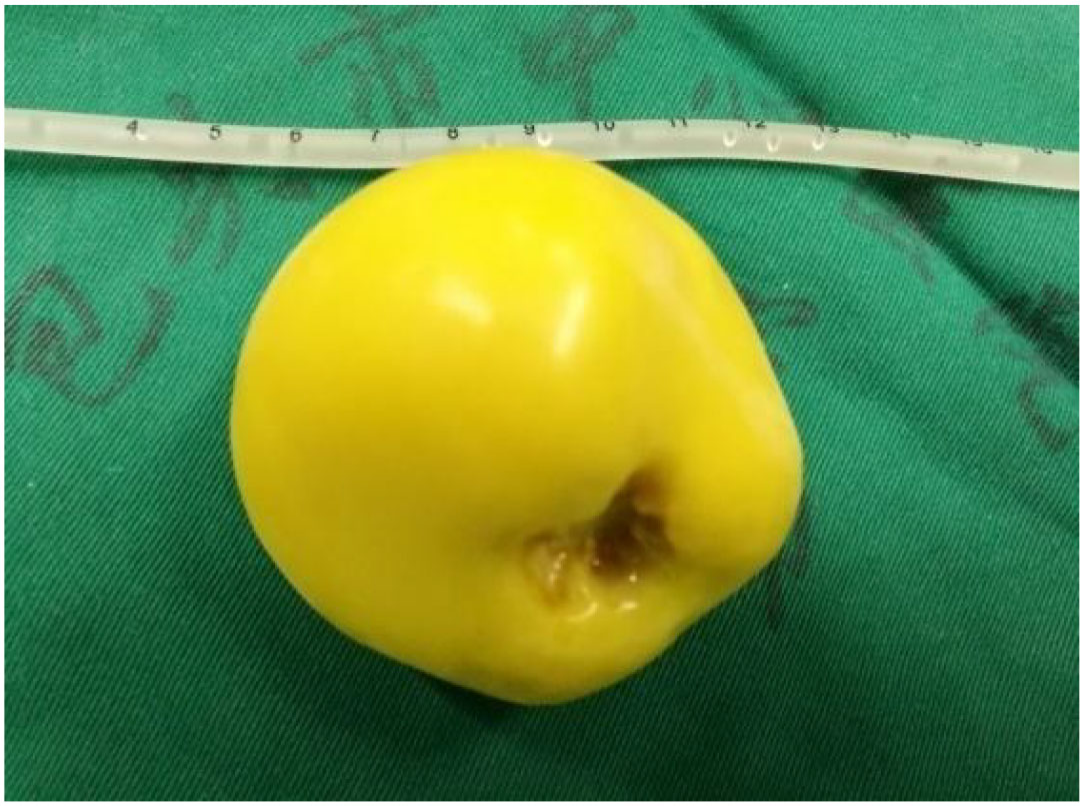
Resected specimen. The peritoneal loose body measures approximately 6 cm in diameter, is yellow and ovoid, with a firm texture and slightly glossy surface.

**Figure 5:**
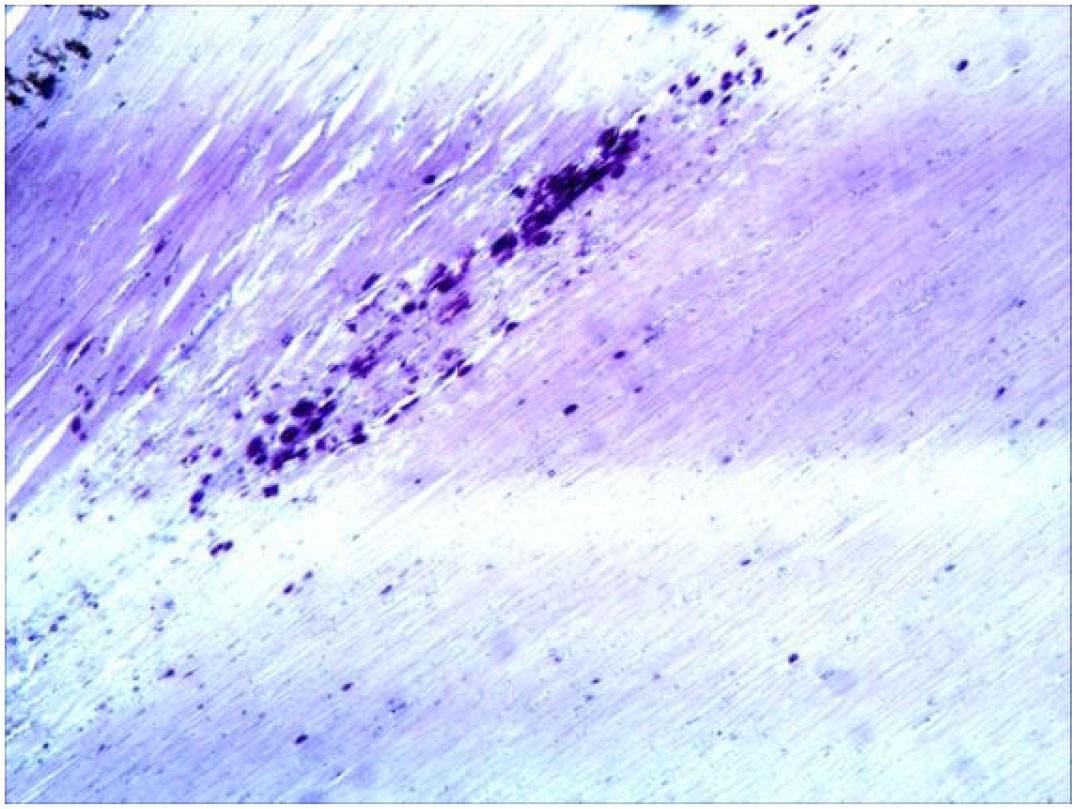
Patchy calcifications are visible within the lesion. (HE ×40).

**Figure 6:**
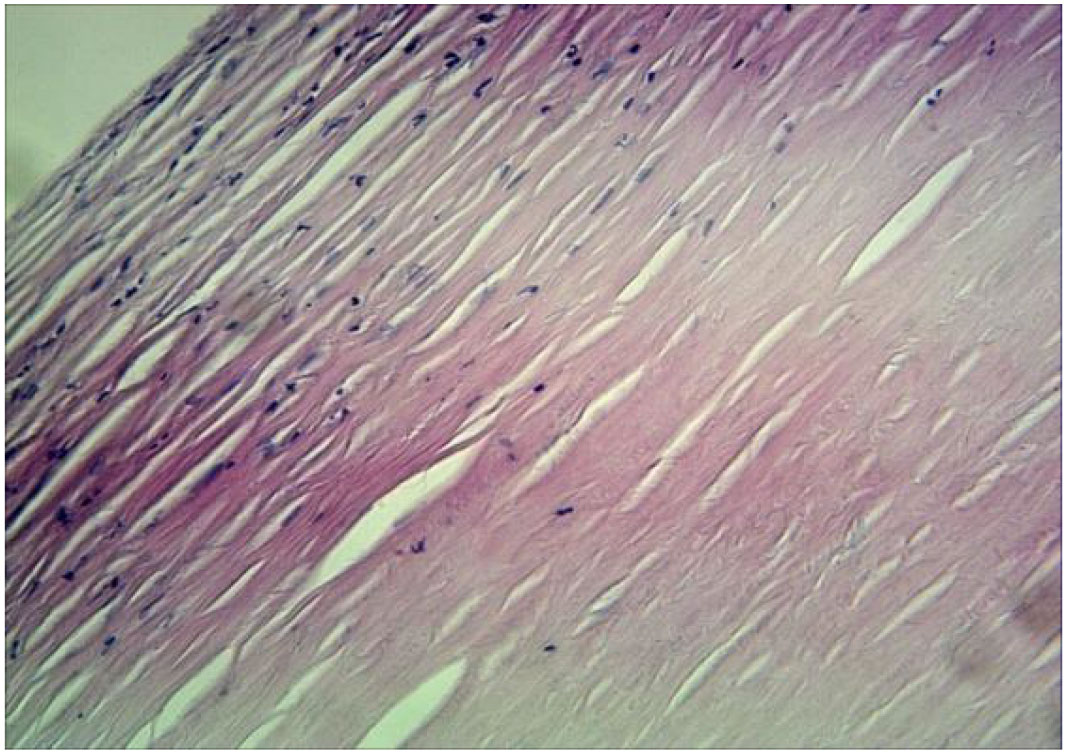
Collagen fibers within the lesion are arranged concentrically, with hyaline degeneration at the margins, appearing uniformly eosinophilic and acellular. (HE×40).

The patient, a male aged 82, was admitted due to “upper abdominal pain for 15 days and jaundice for 7 days.” He experienced severe upper abdominal pain radiating to the back, accompanied by urinary frequency. Physical examination: yellowing of the skin and sclera; positive tenderness in the upper abdomen; no tenderness in the lower abdomen; no palpable mass. Computed tomography(CT) findings: an occupying lesion was observed in the pancreatic head, initially considered pancreatic cancer; on the right side of the pelvis was a quasi-round low-density mass measuring approximately 5.5 × 5.6 cm, with a clear boundary and a central high-density calcification focus (**Figures 1**–**3**). No enhancement was seen on contrast imaging, and a calcified gastrointestinal stromal tumor was initially considered. Laboratory results: CEA 3.15 ng/ml, CA19-9 41 U/ml, ALT 272 U/L, AST 208 U/L, total bilirubin 400.1 μmol/L, albumin 49.6 g/L. Preliminary diagnoses: pancreatic cancer, pelvic stromal tumor, obstructive jaundice, and hepatic insufficiency. After detailed discussion, considering the patient’s advanced age of 82, general condition, and the size of the pelvic mass, exploratory laparotomy was planned. Intraoperative findings: a nodular, firm mass approximately 5 × 5 × 3 cm was located in the pancreatic head, with poor mobility and tight adhesion to surrounding tissues. A spherical free mass approximately 6 × 6 × 5 cm in size, yellow in color, smooth-surfaced, and firm in consistency was found on the right side of the pelvis (**Figure 4**). Because of the patient’s poor baseline condition and the pancreatic head mass invading surrounding tissues, palliative surgery was performed. Cholecystojejunostomy and side-to-side jejunojejunostomy were conducted, along with excision of the abdominal mass. The mass was sent for pathological examination. Gross pathology: the mass was a yellow, quasi-round nodule measuring about 6 × 6 × 5 cm, with a smooth surface and firm texture. The cut surface showed yellow caseous-like material peripherally, with a hard, concentrically arranged calcified center, presenting a gritty sensation on palpation. Histologic features: central necrotic adipose tissue surrounded by concentrically arranged collagen fibers, with hyalinization and calcification (**Figures 5**, **6**). The pathological diagnosis was peritoneal loose body. The pancreatic lesion was diagnosed as malignant pancreatic tumor, not otherwise specified (NOS).Postoperatively, the patient’s liver function and general condition improved, with marked reduction in bilirubin. He was discharged after 2 weeks. As the patient’s Karnofsky Performance Status (KPS) score was <60, he was unable to tolerate chemoradiotherapy; further treatment was planned after improvement of his general condition. Follow-up one month after discharge revealed that the patient had died due to progression of pancreatic cancer.

(This case report was approved with written informed consent from the patient.).

### Literature review

We conducted a PubMed search using the keywords “(giant peritoneal loose body)” OR “(huge peritoneal loose body)” OR “(giant peritoneal loose bodies)” with no time limit, retrieving a total of 41 publications. We excluded irrelevant results and those where the maximum diameter of the loose body was less than 5 cm. Ultimately, the search identified 32 publications reporting on 32 cases of giant peritoneal loose bodies (see **Table 1** for details).

**Table 1:** Clinical characteristics of reported giant peritoneal loose bodies.

S/N	Author and year of publication	Gender	Age	Symptom	Site	Size of peritoneal free body (maximum diameter, cm)	CT examination findings:	Preoperative suspicious diagnosis	Was it considered before the operation that it was a huge peritoneal free body	Treatment	Postoperative discharge time
1	Gupta M, Dhaka SL.(2025) (1)	Male	48	Lower abdominal discomfort and frequent urination	Pelvic cavity	6	Central calcified mass	Calcified dermoid cyst, calcified echinococcosis cyst	No	Laparoscope	2d
2	Agarwal D et al. (2025) (2)	Male	72	Asymptomatic	The pelvic cavity structure has shifted to the right iliac fossa.	6	A centrally calcified mass is present, surrounded by homogeneous soft tissue that shows uniform non-enhancement, with well-defined margins. One year later, an enhanced CT scan revealed a change in the mass’s position, and it still demonstrated no enhancement.	Peritoneal free body free body	Yes	No treatment received	Cannot be obtained
3	Wu K et al. (2024) (3)	Male	68	Acute abdominal pain, difficulty in urination, and dysuria	Pelvic cavity	10	The central area exhibits a triangular high-density configuration, with the outermost layer composed of soft-tissue density and clearly defined margins.	Cannot be obtained	No	Laparotomy	7d
4	Mehammed AH et al. (2024) (4)	Male	61	Chronic intermittent lower abdominal pain, frequent urination and urgent urination	During the CT scan, the mass was located in the pelvic cavity, while intraoperatively, it was found in the left upper abdomen.	6.5	It is a round, well-defined soft-tissue density peritoneal mass with a massive calcification visible in the center. No enhancement is observed on contrast-enhanced scans	Cannot be obtained	No	Laparotomy	Cannot be obtained
5	Ansari N et al. (2022) (5)	Male	55	Chronic abdominal discomfort	One month ago, it was located at the ileocecal region and later found within the pelvis.	7	It is a well-defined mass with a central high-density area surrounded by soft-tissue density.	Mesenchymal tumor of the small intestine	No	Laparotomy	Cannot be obtained
6	Nanno K et al. (2022) (6)	Male	83	Asymptomatic	Near the rectovesical depression outside the abdominal cavity	6	The calcified core and peripheral soft tissues are composed of clear boundaries	Calcific fibrous pseudotumor of the peritoneum	Yes	Laparoscope	5d
7	Allopi N et al. (2021) (7)	Male	79	Chronic abdominal pain	Pelvic cavity, the operation was located in the upper left abdomen	9	A large spherical mass with central calcification	teratoma or non-benign lesion	No	Laparotomy	5d
8	Dhoot NM et al. (2020) (8)	Male	75	Chronic abdominal pain, acute exacerbation	Pelvic cavity	6.2	There is high-density calcification at the center, surrounded by a layer of soft-tissue density, and no significant enhancement is observed on the contrast-enhanced scan.	Iatrogenic foreign bodies or benign pelvic tumors	No	Laparoscope	3d
9	Teklewold B et al. (2019) (9)	Male	50	Intestinal obstruction	Right paracolonic sulcus	7.5	No CT scan was performed	Cannot be obtained	No	Laparotomy	Cannot be obtained
10	Baert L et al. (2019) (10)	Male	53	Chronic abdominal pain and constipation	Pelvic cavity	5.5	The boundaries are clear, it is circular, and there is a high-density core in the center	Foreign Object	No	Laparoscope	Cannot be obtained
11	Guo S et al. (2019) (11)	Male	49	Chronic intermittent lower abdominal pain	Pelvic cavity	5.5	A circular mass with a high density in the center	Cannot be obtained	No	Laparotomy	7d
12	Sahay SC et al. (2018) (12)	Male	50	Dull pain in the lower abdomen, frequent urination and urgent urination	Pelvic cavity	5	No CT scan was performed	Cannot be obtained	No	Laparoscope	Cannot be obtained
13	Obaid M, Gehani S.(2018) (13)	Male	58	Acute abdominal pain with hematuria	Pelvic cavity	6	Mobile mass, high density in the center, no enhancement	A mobile mass in the abdominal cavity	No	Laparoscope	1d
14	Oom R et al. (2018) (14)	Male	64	Asymptomatic	Pelvic cavity	6	A round solid lesion with central calcification and surrounded by soft-tissue density	Pelvic Mass	No	Laparotomy	3d
15	Cojocari N, David L.(2018) (15)	Male	72	Asymptomatic	Pelvic cavity	6.5	A solid mass, with clear boundaries and central calcification	Teratoma	No	Laparotomy	5d
16	Matsubara K et al. (2017) (16)	Male	70	Frequent urination	Pelvic cavity	6.5	No enhanced mass, central calcification	There is strong suspicion that it is a giant PLB However, it cannot be ruled out that there are tumor diseases such as teratoma, leiomyoma or stromal tumor	Yes	Laparoscope	3d
17	Huang Q et al. (2017) (17)	Male	79	Frequent urination	Abdominal and pelvic cavities (2)	10.4 (abdominal cavity) and 7.6(pelvic cavity)	Low-density lesions with clear boundaries and central calcification	Cannot be obtained	No	Laparotomy	3d
18	Rosic T et al. (2016) (18)	Male	73	Frequent urination, difficult micturition and tenesmus	Pelvic cavity	6.6	Soft tissue mass with calcification in the center	A mass of unknown diagnosis	No	Laparoscope	2d
19	Lee KH et al. (2016) (19)	Female	61	Chronic dull pain in the lower abdomen	Pelvic cavity	6	Oval-shaped calcified masses	Subserosal uterine fibroids	No	Laparoscope	Cannot be obtained
20	Elsner A et al. (2016) (20)	Male	52	Chronic intermittent abdomen, constipation	Pelvic cavity	5.2	A spherical mass with central calcification is surrounded by soft tissue and shows no obvious connection to other organs	Foreign bodies left after appendectomy	No	Laparoscope	3d
21	Zhang H et al. (2015) (21)	Male	51	Asymptomatic	Pelvic cavity	5	Low-density lesion, with a complete capsule and two calcifications in the center	Teratoma or intestinal stromal tumor	No	Laparoscope	2d
22	Suganuma I et al. (2015) (22)	Female	35	Asymptomatic	Pelvic cavity	7.5	No CT scan was performed	Uterine fibroid	No	Laparoscope	Cannot be obtained
23	Rubinkiewicz M et al. (2014) (23)	Female	70	Intestinal obstruction, perforation	Pelvic cavity	20	No CT scan was performed	Uterine fibroid	No	Laparotomy	Deceased
24	Sahadev R, Nagappa PK(2014) (24)	Male	52	Symptoms of cholecystitis	Pelvic cavity	7	A well-defined solid space-occupying lesion	Calcified leiomyoma	No	Laparoscope	4d
25	Makineni H et al. (2014) (25)	Male	52	Chronic lower abdominal discomfort and frequent urination	Pelvic cavity	6	A well-defined calcified mass	Calcified leiomyoma	No	Laparotomy	Cannot be obtained
26	Kim HS et al. (2013) (26)	Male	50	Asymptomatic	Pelvic cavity	7.5	The surrounding area is soft tissue, and the center is dense and heterogeneous calcification	Peritoneal calcifying fibrous pseudotumor	No	Laparoscope	5d
27	Sewkani A et al. (2011) (27)	Male	64	Intestinal obstruction	Peritoneal cavity	7	No CT scan was performed	Cannot be obtained	No	Laparotomy	5d
28	Hedawoo JB, Wagh A(2010) (28)	Male	65	Chronic lower abdominal pain, constipation and hemorrhoids	Right iliac fossa	9.5	Unevenly enhanced mass, with calcification in the center and density of peripheral soft tissue	Dermoid cyst	No	Laparotomy	Cannot be obtained
29	Mohri T et al. (2007) (29)	Male	68	Chronic abdominal discomfort and aggravated abdominal pain	Pelvic cavity	7.3 increased to 9.5	Low-density oval-shaped mass with layered calcification	Peritoneal free body	Yes	Laparotomy	7d
30	Takabe K et al. (2006) (30)	Male	68	Intestinal obstruction occurred ten years ago	Lower right abdomen, pelvic cavity (2)	5.8 and 5.2	Two oval-shaped masses with central calcification	Gallstone intestinal obstruction	No	Laparotomy	7d
31	Nomura H et al. (2003) (31)	Male	63	Asymptomatic	Pelvic cavity	5.0	A smooth, circular lesion with two calcifications in the center	Calcified leiomyoma	No	Laparoscope	Cannot be obtained
32	Takada A et al. (1998) (32)	Male	79	Asymptomatic	Pelvic cavities (2)	7 (2)	Two calcified masses	Calcified leiomyoma	No	Laparotomy	Cannot be obtained
33	This case	Male	82	Upper abdominal pain and frequent urination	Pelvic cavity	6	A quasi-circular mixed density shadow with clear boundaries; patchy calcification is seen within it, and no abnormal enhancement is observed.	Gastrointestinal Stromal Tumor	No	Laparotomy	2w

Based on previous cases and the present case, we summarize the following. Giant peritoneal loose bodies occur almost exclusively in males, with only 3 cases reported in females, and the mechanism of formation in females is completely different. The mean age at onset was 62.76 ± 11.90 (35–83) years. Among these, 24 cases were symptomatic, with an average maximum diameter of 7.25 ± 3.01 (5–20) cm. The most common symptoms were abdominal discomfort or abdominal pain (21 cases), mostly chronic (11 cases), followed by urinary irritation symptoms (10 cases). Four cases caused intestinal obstruction, and 1 patient died. The remaining 9 cases were asymptomatic and discovered incidentally. Thirty cases occurred in the pelvic cavity. Prior to surgery, only 4 cases were considered to be giant peritoneal loose bodies in the preliminary diagnosis. Only 1 case did not undergo surgical treatment. Among the others, 15 were removed laparoscopically and 17 by laparotomy. And the average postoperative discharge time was 4.53 days. Notably, 7 cases were complicated by hepatic or renal disease (laboratory abnormalities in liver or kidney function).

## Discussion

Peritoneal loose bodies (PLBs), also known as peritoneal mice, are rare and are usually discovered during surgery, physical examination, or autopsy (1, 16, 18). Most PLBs range in size from 0.5 cm to 2.5 cm, with those larger than 5 cm being referred to as giant peritoneal loose bodies (GPLBs) in the literature (1, 4, 6, 8–10, 13, 15, 20, 21, 25, 29). Since smaller PLBs usually cause no harm to the human body, the present study focuses on GPLBs larger than 5 cm.

### Epidemiology

According to currently reported cases, we found that GPLBs occur predominantly in males; only 3 cases have been reported in females, and their mechanisms of formation were: two cases derived from self-amputated uterine fibroids (22, 23), and one case from a self-amputated right adnexa (19). In other words, all typical egg-shaped GPLBs occur in males. A review of epiploic appendagitis described that the male-to-female ratio of epiploic appendagitis caused by torsion of the epiploic appendage is 4:1 (33). This similarly marked sex difference may further support the hypothesis that GPLBs originate from detached epiploic appendages. This may be related to fat distribution and hormone levels (16). Male visceral fat mass (VFM) is significantly higher than that of females with the same BMI, body fat percentage, and fat mass index (34).The average age at diagnosis of GPLBs was 62.76 years, slightly higher than the previously reported 56.3 years (8), possibly because earlier studies included some PLBs smaller than 5 cm. We excluded the 3 female cases with different potential mechanisms and analyzed only the 30 typical GPLBs that may have originated from epiploic appendages. Pearson correlation analysis showed that patient age was significantly positively correlated with the maximum diameter of GPLBs (r = 0.372, p = 0.043). This result suggests that with increasing age, small PLBs may enlarge into >5 cm GPLBs, causing symptoms and potential severe complications, further indicating the necessity of proactive treatment. However, since the correlation coefficient is of moderate strength (coefficient of determination r² = 0.138), and Spearman rank correlation analysis showed ρ = 0.352 with borderline significance (p = 0.056), age can explain only part of the variability in PLB size. Their occurrence and growth may be influenced by multiple pathophysiological factors. In other words, age may provide the temporal basis for growth, but size is affected by multiple factors rather than being proportional to age alone. Additionally, the sample size is relatively small and may lack sufficient statistical power. Future studies should verify this correlation with additional cases.

### Etiology and mechanism of formation

The pathogenesis and mechanism of formation of PLBs remain unclear. As early as 1863, scholars proposed a formation theory, suggesting that epiploic appendages may undergo torsion and infarction and eventually detach (25).The detached epiploic appendage then undergoes calcification and fibrosis, absorbs proteins from peritoneal serous fluid, gradually increases in volume, and ultimately forms a giant peritoneal loose body, which, under the influence of gravity, falls into the abdominal or pelvic cavity (3, 4, 9).Donald and Kerr produced artificial “peritoneal loose bodies” in guinea pigs, which also supported this view (26). Another report described that, in addition to the GPLB that was removed, part of an epiploic appendage was already calcified with a narrowed stalk, on the verge of detachment, further supporting this hypothesis (32). Meanwhile, based on the pathological characteristics of currently reported cases, classical, egg-like GPLBs are all consistent with an origin from detached epiploic appendages. Non-classical origins include female adnexa and uterine fibroids. The growth rate of PLBs remains unclear. There have been reports of no size change over three years, but also of enlargement from 7.3 × 7.0 cm to 9.5 × 7.5 cm within five years (17, 29). Notably, among patients with typical GPLBs originating from epiploic appendages, 21.21% had concurrent liver or kidney dysfunction. A possible mechanism may be related to changes in the intraperitoneal fluid environment. Studies have shown that approximately 3.8%–4.7% of human serum albumin is exchanged daily between the bloodstream and the peritoneal cavity (35). Various hepatic and renal dysfunctions—such as cirrhosis, hepatitis, nephrotic syndrome, and dialysis-associated ascites—can lead to increased intraperitoneal fluid (36), and protein-rich ascites provides favorable conditions for protein deposition on the surface of loose bodies, potentially accelerating their growth and promoting enlargement (11, 15).Based on this, we propose a hypothesis-generating observation: the formation of GPLBs may be associated with hepatic and renal dysfunction. However, given the limited sample size and the complexity of factors influencing peritoneal loose body growth, current statistical analysis cannot demonstrate that peritoneal loose bodies in patients with liver or kidney dysfunction are significantly larger than those in patients with normal hepatic and renal function. In addition, due to a lack of dynamic observations and comparative studies on the growth process of loose bodies, data are insufficient to confirm that loose bodies grow faster in the presence of hepatic or renal dysfunction. Future studies with larger case numbers are needed to further verify this hypothesis.

### Gross and pathological features

The gross appearance of classical GPLBs is yellow-white and ovoid, firm in texture, with a slightly glossy surface. On cross-section, the outer layer appears white and the core pale yellow, resembling a “hard-boiled egg.” Microscopically, the center consists of necrotic adipose tissue and calcification, surrounded outwardly in concentric layers by eosinophilic fibrous connective tissue, sclerotic collagen, or hyalinized tissue.

### Clinical manifestations

Small PLBs usually do not cause symptoms. As shown in our analysis, patients with GPLBs often present with abdominal pain or abdominal discomfort, accounting for 87.5% of symptomatic cases. Because of gravitational effects, almost all giant loose bodies are located in the pelvic cavity (90.91%), and therefore symptoms of bladder or rectal compression are also common, such as urinary irritation symptoms or intermittent constipation. One past case reported hematuria, suspected to be caused by chronic irritation from the loose body; however, because no further follow-up examinations were conducted, the association between hematuria and GPLBs could not be definitively confirmed (13). The most severe complications reported to date include intestinal obstruction and intestinal perforation (9, 23, 27, 30). One patient with intestinal perforation died due to multiple organ failure resulting from the perforation.

### Diagnosis

Because of their large size, GPLBs are usually not difficult to detect. Their typical imaging characteristics are as follows: CT: usually shows a centrally located high-density calcified focus, with peripheral soft-tissue density, regular quasi-round morphology, and well-defined borders. Contrast-enhanced CT: no enhancement is observed on contrast scanning (2, 4, 8).MRI: both T1 and T2 sequences show a quasi-round low-signal mass, similar to muscular signal intensity (6, 16). PET-CT: some reports show no FDG (fluorodeoxyglucose) uptake (8, 37).Additionally, changes in the position of the mass across repeated imaging studies can serve as an important diagnostic feature of giant peritoneal loose bodies (2, 5, 10, 13). However, due to the low incidence of GPLBs, clinical familiarity with this entity remains limited, and it is often difficult for clinicians to consider that these imaging features represent a giant peritoneal loose body before surgery. Nevertheless, an accurate diagnosis is beneficial for selecting appropriate treatment.

**Differential diagnosis:** 1. Tumors: mesenchymal tumors such as fibroma, lipoma, leiomyoma, gastrointestinal stromal tumor; teratoma.2. Granulomatous diseases: tuberculous granuloma; foreign-body granuloma (including hydatid cyst) (1, 3). Although PLBs are rare in women, they must be differentiated from some gynecological diseases such as uterine fibroids, ovarian metastasis, and ovarian cysts (13, 22). When differentiating from other diseases, attention should be paid to the three key characteristics of GPLBs: (1) High mobility: position changes with changes in body posture; (2) Lack of enhancement: absence of blood supply, thus no enhancement on contrast CT; (3) Tumor markers are usually negative. However, definitive diagnosis still relies on pathological examination.

### Treatment

If small PLBs are discovered during routine physical examination screening, regular follow-up is sufficient. If PLBs are found during surgery, removal is recommended even when small, to prevent enlargement and subsequent complications. For symptomatic giant peritoneal loose bodies, surgical treatment is recommended (20). Whether asymptomatic GPLBs require active treatment remains controversial (5, 6, 8, 15, 16, 20, 26). We recommend that giant peritoneal loose bodies be surgically treated regardless of whether symptoms are present, and that surgery may serve as a direct indication. The reasons are as follows:1. Although abdominal CT/MRI can provide a preliminary diagnosis, other neoplastic diseases cannot be completely excluded; the gold standard remains pathological examination after surgical removal. 2. Serious and even fatal complications caused by giant peritoneal loose bodies—such as intestinal obstruction—have been reported. Their size is also statistically positively correlated with age. Cases have been reported in which patients declined surgery initially but later required surgery due to enlargement and worsening symptoms after five years (29). 3. No postoperative recurrences have been reported (21). Because the intra-abdominal space is relatively large, the capsule of the peritoneal loose body is intact, and there is no significant adhesion to surrounding tissues, the surgical procedure is not technically difficult. Therefore, when giant peritoneal loose body is suspected, we strongly recommend surgical removal regardless of symptoms. Minimally invasive laparoscopic exploration and extraction should be the first-choice procedure.

## Conclusion

GPLB is a rare condition that occurs primarily in elderly males; female cases are extremely uncommon and exhibit distinct mechanisms of formation. GPLB often presents with chronic abdominal pain or urinary irritation symptoms and may lead to intestinal obstruction. Its typical CT appearance is an oval pelvic mass with central calcification. Most originate from torsion, infarction, detachment, and subsequent calcification and fibrosis of epiploic appendages. Its size shows a statistically significant positive correlation with age. Hepatic and renal dysfunction may accelerate its growth. Preoperative diagnosis is challenging and often confused with pelvic tumors; pathological examination remains the gold standard. Given the risks of enlargement and complications, the difficulty of accurate preoperative diagnosis, and the relatively low operative complexity, surgical excision is recommended for lesions ≥5 cm regardless of symptoms, with laparoscopy as the preferred approach due to rapid postoperative recovery. Clinical awareness of this condition should be enhanced to reduce misdiagnosis and optimize management strategies.
